# Derivatives of Plastics as Potential Carcinogenic Factors: The Current State of Knowledge

**DOI:** 10.3390/cancers14194637

**Published:** 2022-09-24

**Authors:** Jacek Baj, James Curtis Dring, Marcin Czeczelewski, Paweł Kozyra, Alicja Forma, Jolanta Flieger, Beata Kowalska, Grzegorz Buszewicz, Grzegorz Teresiński

**Affiliations:** 1 Chair and Department of Anatomy, Medical University of Lublin, 20-090 Lublin, Poland; 2 Chair and Department of Forensic Medicine, Medical University of Lublin, ul. Jaczewskiego 8B, 20-090 Lublin, Poland; 3 Independent Radiopharmacy Unit, Faculty of Pharmacy, Medical University of Lublin, 20-093 Lublin, Poland; 4 Department of Analytical Chemistry, Medical University of Lublin, Chodźki 4A, 20-093 Lublin, Poland; 5 Department of Water Supply and Wastewater Disposal, Lublin University of Technology, 20-618 Lublin, Poland

**Keywords:** microplastic, nanoplastic, carcinogen, environmental factor, carcinogenesis

## Abstract

**Simple Summary:**

Nowadays, micro- and nanoplastic particles can be found almost everywhere, being especially harmful for humans. Their absorption, primarily via inhalation and digestive routes, might lead to a particularly dangerous accumulation of those substances within the human body. Due to the alarming increase in contamination worldwide and excessive production of plastics and synthetic materials, there is an urgent need to investigate the effects of those substances on human health. So far, it has been observed that nano- and microplastics might be extremely harmful, leading to serious health conditions, such as cancers of various human body systems.

**Abstract:**

Micro- and nanoplatics have been already reported to be potential carcinogenic/mutagenic substances that might cause DNA damage, leading to carcinogenesis. Thus, the effects of micro- and nanoplastics exposure on human health are currently being investigated extensively to establish clear relationships between those substances and health consequences. So far, it has been observed that there exists a definite correlation between exposure to micro- and nanoplastic particles and the onset of several cancers. Therefore, we have conducted research using PubMed, Web of Science, and Scopus databases, searching for all the research papers devoted to cancers that could be potentially related to the subject of exposure to nano- and microplastics. Ultimately, in this paper, we have discussed several cancers, including hepatocellular carcinoma, pancreatic cancer, pancreatic ductal adenocarcinoma, biliary tract cancer, and some endocrine-related cancers.

## 1. Introduction

The growth and development of the world population and economy increase human activity in the pursuit of improving living conditions. However, due to poor management, these activities generate xenobiotic pollutants that increase the contamination of global ecosystems [1]. The beginnings of the extensive use of plastics date back to the early 1950s. It is estimated that over 8.3 billion tons of plastic have already been produced, 3/4 of which currently constitutes waste [2,3]. Despite such a short history of ‘salutary’ plastic, this material can now be traced everywhere (even to the North Pole) and currently, we can talk about the global problem of plastic pollution (GPPP).

Nowadays, we cannot imagine a world without plastic, even though we have only started using it recently [2]. In the past few years, over 360 million metric tons (Mt) of plastic have been produced worldwide each year [4], 40% of which is single-use packaging [5,6]. Most of it is discarded into the environment [7,8]. Geyer et al. (2017) estimate that if the current trends in production and economy are maintained, by 2050, there will be 12,000 Mt of plastic in the environment [2]. Other more drastic estimates are that if we do not stop GPPP, one day, we will have more plastic than fish in the sea (considering the mass) [6]. The authors agree that plastic pollutants are delivered to the marine environment via rivers from land, where they are generated [9,10,11]. Numerous models estimate that up to 30 Mt of plastic can end their ’life-cycle’ in the earth’s aquatic environment [12,13,14]. Plastic-based products are practically non-biodegradable in the natural environment; however, as a result of biological, physical, and chemical processes, they transform into smaller particles defined as microplastic (MP) and nanoplastic (NP) [15,16,17,18]. The small size of the MP and NP makes it easy for these to be absorbed by the marine fauna [19], after which they bioaccumulate and are biomagnified [20,21], further enriching higher food chains [22,23].

**Figure 1:**
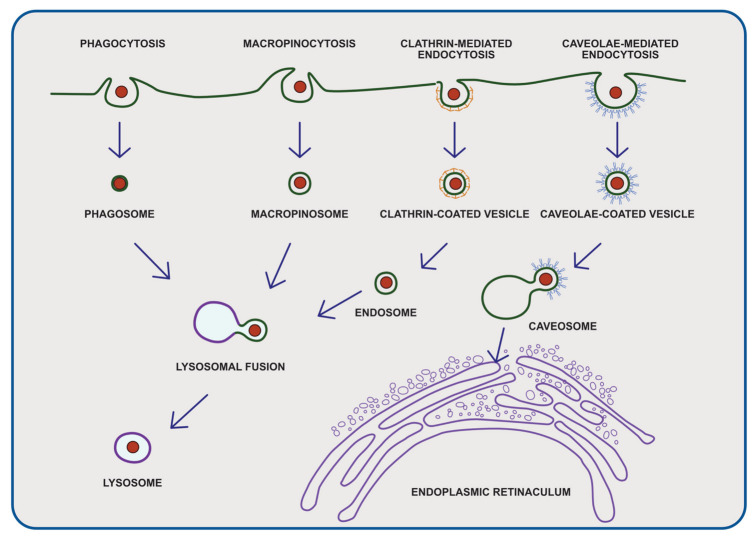
Four routes of cellular uptake of plastic particles: (1) phagocytosis, (2) micropinocytosis, (3) clathrin-mediated endocytosis, (4) caveolae-mediated endocytosis. After entering the human body, micro- and nanoplastics can interact with various cells; the number of absorbed particles depend on several factors, including the size, surface, charge, and chemistry of the encountered cells to which micro- and nanoplastics are absorbed.

GPPP can have adverse effects in all three ecosystems, which can ultimately lead to economic damage and, more importantly, can affect human safety and health to a large extent [24,25,26,27,28,29]. This would result, for instance, in the consumption of contaminated food through which MP and NP enter the human body (Figure 1) [30,31,32,33,34].

Enyoh et al. (2019) report that when consuming fruit and vegetables, exposure to plastic reaches up to 80 g MP per day [35]. MP and NP enter the human body even when consuming drinking water [36] and during normal breathing [37]. This year, it has also been reported that microplastic has even been detected in the human placenta [38]. In addition, there have been reports of finding MP in human stool [39] and human colectomy specimens [40]. The fact is that the actual human exposure to MPP and NP is still debatable because of various methodological limitations of the studies related to this topic. Domenech and Marcos (2021) pointed out that MP and NP might be absorbed via ingestion, inhalation, and dermal contact; however, due to the lack of robust and standardized protocols, the detection and the total burden of particles found in the human body might be highly underrated [41]. Zarus et al. (2020) also identified data gaps in the existing literature, namely the lack of characterization of plastic particles and fibers smaller than 10 μm [42]. Thus, in recent years, GPPP has become a subject of increasing concern, resulting in large-scale research [43,44,45].

## 2. Aim of the Review and Search Strategy

The main objective of this study is to highlight the potential findings related to micro and nano particles in terms of human health and to investigate the possible relationship between the exposure to those particles and potential carcinogenic effects. A thorough search of the articles was conducted, related to the topic of this paper, via the use of the following databases: PubMed, Scopus, and Web of Science by two identifications in June 2022. The first identification aimed to search and identify the currently reported types of cancers that could be potentially induced by MP and NP; the search strategy was as follows: (microplastic OR nanoplastics) AND (cancer OR tumor OR carcinogenesis OR tumorigenesis). Following the revision of the articles and papers, we chose the following cancers to be discussed in our paper: biliary tract cancer, hepatocellular carcinoma and pancreatic cancer. The second identification of the literature was performed using the following search strategy: (microplastic OR nanoplastics) AND (biliary tract cancer OR hepatocellular carcinoma OR pancreatic cancer) OR (leukemia). There were no restrictions regarding the year of the publication and we chose only the articles written in English.

## 3. Definition and Classification of Micro- and Nanoplastics

MP consists of particles that range in size from 0.1 to 5000 µm, and NP ranges from 0.001 to 0.1 µm [46], which are water-insoluble solid particles or polymer matrices of regular or irregular shape [47]. Primary MP particles are the ingredients of cleaning agents [48], toothpastes [49], scrubs [50], hand soaps [51] or biomedical products [52], and the secondary particles are those obtained by fragmentation processes under the influence of UV radiation or physicochemical processes, such as pH or salinity [53,54,55,56,57,58,59]. These include household garbage, plastic films or emissions from vehicles [60,61,62]. The shape of the MP depends on the destruction processes, a lifetime in the environment and the original form of the material; therefore, we can identify fibers, balls, films, granules, or flakes [63,64,65]. Qi et al. (2020) reports that the harmfulness depends on the size and shape of the MP [66]. The negative effect of microplastics is expressed in physical effects, mainly in the concentration of microplastics and chemical effects related to the chemicals contained in them.

### 3.1. Microplastic Components

MP is most often composed of polypropylene (PP), polystyrene (PS) or polyethylene (PE) [67]. Polypropylene is relatively chemically resistant. It has a crystalline structure with a high level of rigidity. Its hardness is due to the presence of methyl groups in the molecular chain. PP is characterized by a high melting point [68]. Polypropylene comes in three types, as a PP homopolymer (HPP) containing propylene monomers in a semi-crystalline solid form, as a random copolymer (RCP), containing, apart from propylene, a small addition of ethylene as a comonomer, and an impact copolymer (ICP), which contains a mixture of HPP and RCP with ethylene content of about 50%.

Polystyrene is highly thermoplastic. It is used for the production of toys, toothbrushes, CDs or polystyrene, itself. Polystyrene is also used in the production of food containers. It is formed as a result of the polymerization of styrene components [67,69]. PS is thermostable, and therefore provides excellent thermal insulation as it is also chemically resistant. 

Polyethylene consists of long chains formed by ethylene monomers. It is a stable polymer [70]. In addition, it is an excellent electrical insulator, characterized by high strength and flexibility [71]. It is the most popular plastic [72]. We can distinguish between high-density polyethylene (HDPE), low-density PE (LDPE) and linear low-density PE (LLDPE) [73].

### 3.2. Industrial Classification of Chemical Contaminants

Campanale et al. (2020) divides chemicals into additives with polymer raw materials and those that are adsorbed from the environment [47]. Additives are substances that give plastic the desired properties, and they include inert or reinforcing fillers, plasticizers, antioxidants, UV stabilizers, lubricants, dyes and flame retardants [74]. Inert fillers provide strength, improve flow and shrinkage of plastics and include asbestos, glass, rutile, silica, talc, clays, chalk, aluminum oxide, soot and carbon nanotubes [75]. Plasticizers are placed between the chains of molecules, ensuring an improvement in elasticity, mobility and plasticity [76]. Stabilizers ensure thermal and chemical stability and they consist of organic or inorganic salts of barium, lead and cadmium [47]. Dyes are used to give color to the polymer. They are divided into inorganic containing heavy metals and organic ones containing phthalocyanine, azo, and anthraquinone groups and many other chromophores [77]. Calcium and magnesium stearate are components of lubricants and adhesives, and their addition facilitates the flow of the substance. Flame retardants contain chlorine, bromine, phosphorus and aluminum hydroxide to protect the material in the event of fire [47]. However, many of these additives, are toxic [74].

### 3.3. Chemical Classification of Main Chemical Contaminants

#### 3.3.1. Heavy Metals

Heavy metals are metals with a density greater than 5 g/cm^3^ [47,78]. They access the environment as a result of human activities and may negatively affect the ecosystem [79,80]. Heavy metal pollution is greatest in the vicinity of harbors and marinas, which correlates with large amounts of MP being traced there [20]. They adhere to the MP surface and interact with the environment. In recent years, the toxicity of heavy metals that adhere to microplastics has been studied and the reports obtained prove high concentrations of Cr, Ni, Fe, Co, Cd, Al, Zn, Mn, Cu. Despite numerous reports on the uptake of a significant amount of toxic metals on the surface of microplastics in the marine environment, there are only limited data for soil and air environment [37,78,80,81] toxicity.

Imran et al. (2019) report distressing conclusions, indicating that mercury, lead, zinc, copper and cadmium can induce co-selection of antibiotic resistance in bacteria [82]. Moreover, Richard et al. (2019) report that MP is an excellent vector for transferring genes between phylogenetically separate microorganisms, which may pose a real threat to human health [83].

#### 3.3.2. Organic Components

Polychlorinated biphenyls (PCBs) are formed by the fusion of 1 to 10 hydrogen atoms with chlorine atoms in the biphenyl ring [84]. A total of 113 of the 209 possible PCB congeners exist in the environment [85]. In 1972, the first report of PCB absorption on polystyrene at a concentration of 5000 ng/g appeared [86]. Currently, there is an agreement on their environmental increase in concentrations of up to 18,700 ng/g [78]. The numbers of PCB congeners commonly reported in the literature are 52, 101, 118, 170, 138, 153 [78].

Polycyclic aromatic hydrocarbons (PAHs) are compounds whose structure consists of many aromatic rings. On the MP surface, the presence and quantification was demonstrated for 3-methylphenanthrene, 9-methylphenanthrene, 2-methylphenanthrene, 1-methylphenanthrene, pyrene, benzo[b]fluorene, 2-methylpyrene, 1-methylpyrene, benzo[b]fluoranthene, chrysenic, 4-methylpyrene, benzo[j]fluoranthene, benzo[a]anthracene, benzo[k]fluoranthene, benzo[e]pyrene, benzo[a]pyrene, indeno[1,2,3-cd]pyrene, benzo[ghi]perylene, 4H-cyclopenta[def]phenanthrene, coronene, perylene, fluoranthene, phenanthrene, anthracene [78,87,88,89]. Due to their high hydrophobicity, which correlates with low water solubility, PAHs may easily settle in soil and sediments [90]. It is worth adding that the concentration of phenanthrene on polystyrene was higher than in the surrounding water reservoirs [8]. This may lead to the increase in the toxicity of MP. Moreover, it is reported that a higher concentration of PHA is observed on the discolored MPs rather than on new ones [78].

Organochlorine pesticides (OCP) are chlorinated derivatives of hydrocarbons. They are synthetic pesticides widely used in the chemical industry and agriculture [78,91]. OCPs are characterized by high persistence in the environment and are classified as persistent organic pollutants [92]. OCPs have highly lipophilic properties; therefore, it is suspected that they can accumulate in plants with greater predominance of lipids and lower water content [93]. The literature mentions chlordanes, cyclodienes, mirex, hopanes: natural substances, hexachlorobenzene and dichlorodiphenyltrichloroethane and its derivatives [78].

PBDEs are ether diphenyls in which bromine replaces the ring hydrogen atom at 1–10 position. A total of 206 congeners are possible. Under the influence of natural physico-chemical factors, they can undergo deprotonation, which, although rare, results in the formation of highly toxic polybrominated dibenzenofurans, brominated phenols and bromobenzenes, [94]. The most common and present in high concentrations is the BDE-209 congener.

Perfluorinated compounds (PFCs) are those with a hydrocarbon backbone in which all the hydrogen atoms are replaced with fluorine atoms. This ensures high thermal and chemical stability [78,95]. This group includes fluorotelomer alcohols, fluoropolymers and perfluorinated carboxylic acids. The best described, from the toxicological point of view, are perfluorooctane sulfonic acid and perfluorooctanoic acid [95]. Further research is needed to determine the degree of their adsorption on the MP [78].

Bisphenol A (BPA), a diphenylmethane derivative, is a component of the monomer in polycarbonate and is used in the production of food and drink containers [96]. Nevertheless, it is highly unstable, which results in easy leaching, which correlates with its abundant presence in the aquatic environment. Despite this, there is still insufficient research on the adsorption of BPA in MP [74]. A cause for concern is BPA’s structural similarities to hormones, which allows mimicking and interference with the endocrine system [97].

As for aliphatic hydrocarbons, there are no literature reports regarding their toxicity. Short-chain hydrocarbons are easily degradable, but those with higher chain groups are less degradable. However, this may help to diversify the sources of organic matter. It should also be noted that there are insufficient data on the adsorption of aliphatic hydrocarbons in MP [78].

Derivatives of octylphenols (OP) and nonyphenols (NPs) are the most common detergent additives, which obviously results in their increased presence in the aquatic environment [78]. Their action may disrupt the hormonal balance, which makes them highly toxic [98,99,100]. However, there are only a few studies on their adsorption in MP [78].

Phthalates are ester derivatives of 1,2-benzenedicarboxylic acid. Phthalates are strongly lipophilic, which makes them firmly sorbed in the soil, which also allows us to conclude that they have a high sorption capacity in MP, but this phenomenon has not been investigated yet. Many phthalates, in particular (di (2-ethylhexyl) phthalate (DEHP) and dibutyl phthalate (DBP)), are toxic and subject to product concentration restrictions [47].

## 4. Exposure Routes

Bearing in mind the abundance and environmental persistence, exposure of humans to micro- and nanoplastics is inevitable. Humans are liable to small plastic particles via the following three routes: oral (intake of contaminated water and food), respiratory and dermal (via skin cleansers/facial scrubbers).

### 4.1. Gastrointestinal Tract

The primary plastic entry point into the human system is the gastrointestinal tract [30,33]. Involuntary plastic ingestion by humans may happen via the food chain with consumption of contaminated food and drinks [101]. Contamination may occur also through the migration of nanoplastic particles from the packaging materials into food products [102].

According to the literature, micro- and nanoplastic particles are present in widespread marine products, including fish, mussels, lobsters, oysters, sea cucumbers, and scallops [103]. Plastic particles present in seafood are mostly smaller than 300 μm [101] and can accumulate organic pollutants and pesticides, including PCBs, PAHs, DDT, and PBDEs [102,104]. For Europeans, who consume seafood quite frequently, the estimated plastic consumption is 11,000 particles per year [105]. Micro- and nanoplastic fibers are also present in other foods, including beer, honey, table salt, and sugar [106,107,108]. Additional exposure results from drinking water in plastic bottles [109,110,111]. In bottled mineral water from nine countries, the contamination with microplastic was estimated from 0 to over 10,000 particles/L (size range of 6.5–100 μm) [112].

Shwabl et. al. identified a median of 20 microplastic particles per 10 g of human stool samples, which confirms their involuntary ingestion [39]. Based on animal models, plastic particles in certain size fractions (0.1 and 150 μm) can move across the mammalian gut into the lymphatic system via endocytosis using M cells of Peyer’s patches [113]. Walczak et al. (2014) determined that the internalization and translocation of polystyrene nanoparticles in human intestinal cell models depend on the size and chemical composition of the particle [114]. However, the information regarding the degree of uptake and translocation of micro- and nanoplastic from the human gastrointestinal system still seems to remain insufficient.

### 4.2. Respiratory System

Another entry point of plastics into the human body is via the respiratory system [37]. The sources of airborne microplastic include synthetic fabrics from clothing, rubber tire erosion, household objects, building materials, landfills, abrasive powders and 3D printing [114,115,116]. According to Dris et al. (2017), up to 33% of household dust fallout is microplastic with polypropylene being predominant, and cellulose [117]. High microplastic concentrations indoors may be explained both by examining numerous sources of plastic, including household objects and synthetic textiles, and the mechanisms involved in their dispersion, such as ventilation rate, airflow, room partition, as well as climatic conditions [81,118].

In the lungs, a very thin tissue barrier, smaller than 1 μm, separates the lumen of the alveoli from the bloodstream [119]. Nanosized particles bear the potential to penetrate the capillary blood system and be distributed throughout the human body [120]. In vitro studies have shown that nanoplastic particles are absorbed by alveolar epithelial cells [121,122]. Occupational studies that have investigated the exposure to synthetic polymers [123,124,125,126], combined with the recent detection of MPs in airborne samples, point to a possible risk for human exposure via inhalation [116].

### 4.3. Skin

The last route of exposure of plastics into the human body is through the skin. Skin constitutes the outer shell of the body that protects the body against heat, light, injury, and infection. Skin can come into contact with plastic particles, especially when cosmetic products containing nanoplastic are used [127].

The stratum corneum of the skin is a solid barrier composed of corneocytes, surrounded by hydrophilic lipids that prevents penetration of hydrophobic agents; hence, significant absorption of nanoplastic particles through the skin is not expected [128]. According to the studies conducted on a porcine skin tissue model, the polystyrene particles with diameters of 20–200 nm were unable to penetrate below the stratum corneum into deeper layers of the skin [129,130]. Nevertheless, there are no recent studies that have examined the ability of nanoplastics to penetrate the skin. Therefore, the data available from studies using nanoparticles may be used to evaluate the ability of nanoplastics to cross the skin barrier.

Some additional factors may increase the nano-permeability of the stratum corneum. According to Mortensen et al. (2008), exposure to UV radiation disrupts the expression of tight junction-related proteins (zonula occludens-1, claudin-1, and occludin), which increases the skin penetration of nanoparticles (carboxylated quantum dots) in mice models [131]. In addition, certain ingredients of skin lotions (e.g., urea, glycerol, and α-hydroxyl acids) enhance the penetration of nanoparticles into the skin [132].

## 5. Micro and Nanoplastic in Organs of the Systems

### 5.1. Cardiovascular System

With respect to the effects on the cardiovascular system, a study on developing zebra fishes proved that the main site of accumulation of nanoplastic particles was the pericardial sac. In the studied group, the pericardial accumulation occurred only in higher concentrations of nanoplastic (1 ppm and 10 ppm). Moreover, there was an observed dose-dependent decrease in heart rate by 5–10% in all zebrafish (Danio rerio) larvae groups, from 0.1 ppm to 10 ppm concentrations [133]. The accumulation of nanoplastic in the pericardium was also demonstrated by Veneman et al. (2017), who directly injected the plastic particles into the yolk sac of zebrafish embryos [134]. Pericardial edema decreased cardiac output, and inhibition of the subintestinal angiogenesis was confirmed by Sun et al. (2021) in zebrafish embryos cultured with 50–200 μg/mL nanoplastic particles [135]. Investigation performed on marine medaka (Oryzias melastigma) also ascertained the decreased heart rate, as well as decreased body length, after the exposure to 20 μg/L polystyrene microplastics [136]. In studies by Li et al. (2020), the elevated cardiac damage markers (troponin I and creatine kinase-MB) were detected in 6-week-old rats exposed to 0.5 μm polystyrene particles over 90 days [137,138].

### 5.2. Gastrointestinal System

Gut epithelium is the largest mucosal surface of the human body. It is the first line of defense of the intestinal lumen and the internal environment. The exposure to microplastic particles caused deformation and disorder of intestinal epithelial cells in Artemia parthenogenetic larvae and earthworms, which compromised the integrity of the intestinal barrier [139,140]. In mice exposed to 5 μm microplastic, Luo et al. (2019) detected a significant decrease in transcription levels of tight junction proteins (Zo-1 and Claudin-1) [141].

Except for damaging cell-to-cell integrity of the intestinal wall, many studies have reported the damage to the mucus barrier in response to plastic exposure. Kang et al. (2021) reported an increased mucus ratio in the gut of marine medaka after exposure to 50 nm and 45 μm plastic particles [142]. In goldfish larvae, following the exposure to both nano and microplastic (70 nm and 50 μm), the destruction of the intestinal mucosa and the loss of the submucosal structure were observed [143]. In a series of studies conducted on mice exposed to 5 μm microplastic, a significant decrease in mucus secretion was observed. Moreover, the expression of genes related to mucin secretion (such as Muc1, Muc2, Muc3, Klf4, Meplin-β, and Retnlb) decreased [144,145].

Up to 100 trillion symbiotic microbes inhabit the human gut, which is known as the gut microbiota. The active gut microbiota can promote digestion, regulate the expression of genes, and affect human immune and metabolic processes by the production of a large number of substances, including short-chain fatty acids, vitamins, and health-beneficial products, such as anti-inflammatory, analgesic, and antioxidant products [146]. Studies have reported that plastic particles can affect the diversity and composition of gut microbiota. In medaka, exposure to microplastic particles has decreased the abundance of Bacteroides and caused significant changes to microbiota at the phylum and genus level [142,147]. Significant biological disorders and changes in the relative abundance of microorganisms in response to micro- and nanoplastic exposition were observed also in juvenile guppies and zebrafish larvae [148,149,150]. In mice exposed to microplastic for 6 weeks, a decrease in Firmicutes, Actinobacteria, and Proteobacteria was observed [144]. According to Luo et al. (2019), exposure to 5 μm microplastic during pregnancy and lactation of mother mice increased the abundance of Actinobacteria and Epsilon bacteraeota in the offspring [141].

The impact of plastic on the gut microbiota has not yet been investigated; however, it may provide significant evidence on the diseases that result from microbiome dysbiosis, including diabetes, cardiovascular disease, and colon cancer [151,152,153,154].

### 5.3. Reproductive System

The reproductive impact of micro- and nanoplastic has been investigated in a variety of organisms. The main target of plastic particles seems to be the embryo life cycle. It is a vulnerable stage when harmful factors may significantly disrupt the development of the organism. The plastic particles larger than 100 nm are not able to migrate through chorion pores [155]. Instead, they adhere to its surface and reduce the embryo’s oxygen absorption, resulting in changes in heart rate, which delay hatching [156]. Some nanoplastic particles that pass the barrier accumulate in the yolk sack [157,158]. Duan et al. (2020), in a study on zebrafish larvae, revealed that nanoplastic from embryonic development may accumulate in the brain, gills, blood, liver, and the digestive tract [155]. Exposure of oyster larvae to 1–5 μm microplastic inhibits their swimming ability and causes severe developmental impairment [159].

**Figure 2:**
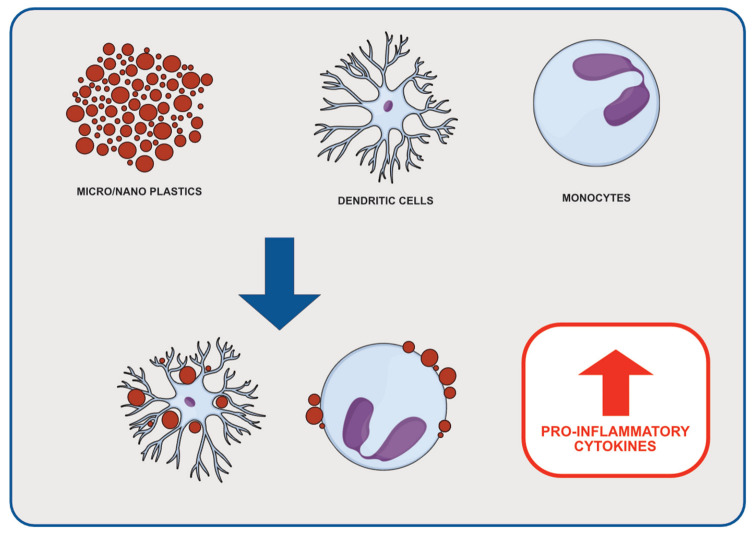
Effects of micro- and nanoplastic on inflammatory cells. Micro- and nanoplastics binding to cells such as dendritic cells or monocytes might stimulate the release of pro-inflammatory cytokines.

Except for influencing the embryo and offspring development, nano and microplastic may impair reproduction at the stage of gamete formation and maturation by disorders in the hypothalamic–pituitary–gonadal axis [156]. Moreover, sperm cells may be damaged by oxidative stress and inflammation caused by plastic particles (Figure 2) [160,161,162].

### 5.4. Nervous System

**Figure 3:**
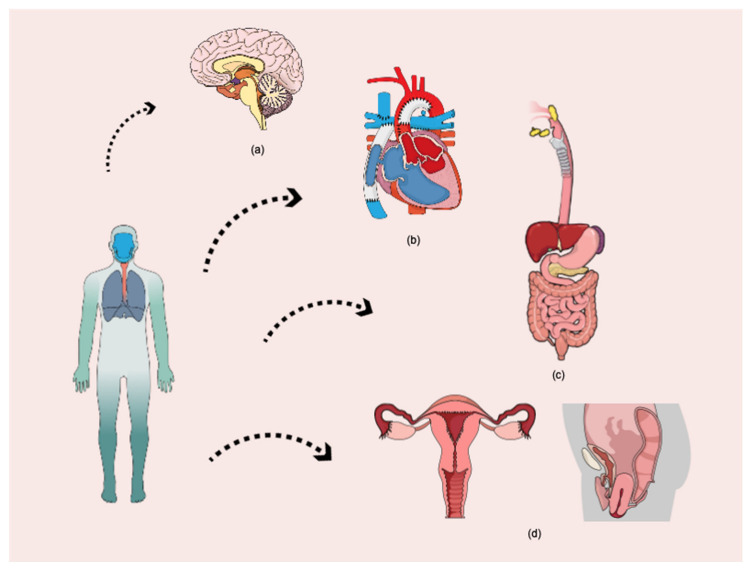
Major sites of micro- and nanoplastic particle accumulation in the human organism: (**a**) nervous system, (**b**) cardiovascular system, (**c**) gastrointestinal system, (**d**) reproductive system.

The presence of micro- and nanoplastic in the nervous system may exert a toxic effect that is caused mainly by oxidative stress and inhibition of the AchE enzyme [163]. AchE is responsible for the degradation of acetylcholine, hence, for normal nerve signal transmission [164]. Its inhibition may lead to overexcitation of the neurons and neurological disorders. Nanosized particles are potentially more neurotoxic, as smaller sizes may more easily penetrate the blood–brain barrier [165]. The zebrafish treated with nanoplastic exhibited behavior changes, such as disturbance of locomotion, low food intake frequency, and depression [163,166]. Microscopic examination of the brain showed inflammation, necrosis, and degradation of neurons (Figure 3) [163].

## 6. Cytotoxic Effect of Micro- and Nanoplastic

### 6.1. Inflammation

Both animal studies and in vitro trials showed that the accumulation of plastic particles leads to inflammation. Micro- and nanoplastic particles, recognized as foreign agents by the immune system, may induce the immune response and ultimately cause host toxicity [167]. In human gastric adenocarcinoma, lung carcinoma, leukemia, and histiocytic lymphoma cells, polystyrene nanoparticles increase the expression of IL-6 and IL-8 genes [168,169,170]. In mice, the intake of polyethylene microplastic results in the expression of toll-like receptor 4 (TLR4), Jun proto-oncogene, AP-1 transcription factor subunit (AP1), and interferon regulatory factor 5 (IRF5) in the intestines [171].

Macrophages are the main phagocytic cells that uptake plastic particles. Prietl et al. (2014) found that carboxylated nanoplastics impair the phagocytic functions, including chemotaxis, cytokine release, and nitric oxide production of monocytes and macrophages [170]. An investigation into how plastic particles impact the polarization of human macrophages showed that amino-functionalized and carboxylated nanoplastic inhibited the release of IL-10 and expression of CD163 and CD200R receptors in M2 cells [172].

Hwang et al. (2019) found that at high concentrations, nanoplastic particles stimulated the immune system and enhanced potential hypersensitivity of murine macrophages (Raw 264.7), and in the human dermal fibroblast (HMC-1) via an increase in the levels of cytokines (IL-6 and TNF-α) and histamines [173]. However, Stock et al. (2019) revealed that polystyrene microparticles did not interfere with the differentiation and activation of the human macrophages [174].

### 6.2. Oxidative Stress and Apoptosis

Oxidative stress can induce cell apoptosis, which is considered the key pathway of micro- and nanoplastic toxicity. The source of oxidative stress may be a large surface area of plastic particles, oxidizing the species (e.g., metals) stuck to their surface, and induction of an inflammatory response [175,176]. Moreover, plastic particles have various functional groups and chemical bonds (such as phenyl groups, amide groups), which may be related to oxidative stress [177,178,179]. Nanoplastic particles induce stronger antioxidant responses compared to micro-plastic [142].

Many in vitro studies have identified increased oxidative stress and apoptosis in human cells, including hematological cells, alveolar epithelial cells, lung cancer cells, and colon carcinoma cells, following polystyrene exposure [180,181,182,183,184]. The production of reactive oxygen species induced by cationic polystyrene nanoparticles caused aggregation of misfolded proteins and autophagic death of macrophages (RAW 264.7) and lung epithelial cells (BEAS-2B) in mice [185,186]. Furthermore, in vivo studies in Wistar rats have shown ROS-induced cardiotoxicity, causing apoptosis of cardiomyocytes and structural damage to the myocardium [138,187].

### 6.3. Toxic Compounds from Plastic

Besides particle toxicity, micro- and nanoplastic could also present chemical and biological risks. Plastic has numerous additives that improve its properties. The Endocrine Society outlines approximately 144 hazardous chemicals or chemical groups that are used as additives [188]. These substances may leach from the plastic matrix inside the organism. The most investigated species are bisphenol A (BPA), vinyl chloride (VC), and benzyl butyl phthalate (BBP) [189,190,191]. In recent years, it has been shown that exposure to BPA during pregnancy reduces the survival rate and birth weight of offspring [192]. It also exerts a hormonal effect, as it mimics the estrogenic hormone, thus increasing the likelihood of developing carbohydrate disorders and cardiovascular disease [193,194,195].

Plastic particles can also absorb substances such as metals, PAHs, phthalates, PFAAS from the surrounding environment [47,191,196,197,198]. Although these substances are either not absorbed or degrade rapidly in the human body, plastic particles facilitate their penetration and make them stay in the body longer [199]. The adverse effects include acute inflammation of the liver caused by plastic-associated metal [200] or carcinogenicity of PAHs [196].

## 7. Potential Carcinogenic Effects of Micro-/Nanoplastics and Their Derivatives

Heavy metal components are well documented in creating and sustaining plastics through their delivery in commercial products, stabilizers, biocides and pigments. A slew of carcinogenic, neurotoxic, and hormone-disrupting chemicals are common constituents and waste products of plastic manufacture, and they invariably make their way into our environment via water, land, and air pollution. Vinyl chloride (in PVC), dioxins (in PVC), benzene (in polystyrene), phthalates and other plasticizers (in PVC and others), formaldehyde, and bisphenol-A, or BPA, are some of the most well-known substances (in polycarbonate) [193]. The plastic industry emits a large amount of harmful gaseous pollutants into the air, including carbon monoxide, dioxins, and hydrogen cyanide. These gases damage the air and their presence at large concentrations in the air is harmful to both human and animal health.

In the twenty-first century, the world’s population took a stand and introduced plastic recycling, which has just recently become a significant solution to the planet’s homeostasis, with plans to curb and prevent further damage. A study based around a plastic recycling area in Northern China produced significant results related to toxic heavy metals from the soil samples. Many plastics yield small, yet potentially toxic elements, such as Cd and Hg, which can easily sediment in nearby lakes, rivers and soils [201]. Both carcinogenic and non-carcinogenic effects can impact human health; although analysis on their exposure needs to be congruent with their clinical findings, healthcare practitioners should be aware of their local recycling plants. Exposure can occur from ingestion, dermal contact, as well as via inhaling. However, occupational workers present higher risks of exposure to the toxic elements, potentially altering and inhibiting their metabolic functions.

### 7.1. Endocrine-Related Cancers

Endocrine disruptors are substances that can alter and interfere with endocrine functioning, also known as hormonally active agents, endocrine disruptive chemicals, or endocrine disrupting compounds [202,203,204]. Cancerous tumors, birth abnormalities, and other developmental diseases can result from these changes [204]. Endocrine disruptors, which can be found in a wide range of consumer and industrial products, might interfere with the synthesis, secretion, transport, binding, action, or elimination of natural hormones in the body and are responsible for development, behavior, fertility, and the maintenance of homeostasis, as well as the onset of malignant and non-malignant diseases [204,205,206,207]. Fucic et al. (2018) has outstandingly highlighted specific cancers, mentioning estrogen/testosterone coupling receptors, for instance, colorectal, pleural, and bladder cancer [208]. Both construction and plastic sector workers had a much higher rate of testicular cancer [207,208,209,210,211].

### 7.2. Biliary Tract Cancer

Ahrens et al. (2007) constructed a detailed multi-center study within six European countries. The findings indicate some association between workplace exposure to endocrine-disrupting agents and the risk of extrahepatic biliary tract cancer in men, notably in the extrahepatic bile duct and ampulla of Vater. Polychlorinated biphenyls may pose a significant threat [212]. The collected data point to a raising of the level of complexity due to numerous microplastic derivatives around endocrine disruption; the researchers themselves pointed out limited data collection and restricted specificity for both gallbladder carcinoma and liver cancer. The results seem very informative; however, the need for further clarity and precision is indispensable. The researchers also emphasize that extrahepatic biliary tract cancer risk was elevated among those individuals who had been exposed to endocrine-disrupting chemicals with recognized estrogenic activity (alkylphenols, PCB, bisphenol A), with PCB exposure being statistically significant [212]. Based on this strong finding, with the alignment of pathophysiology-related carcinogenic attributes, estrogenic receptors may be a key feature to consider in future studies.

### 7.3. Hepatocellular Carcinoma

The development of hepatocellular carcinoma (HCC) is considered an ambiguous manifestation that takes years to develop, but can be enhanced via toxic substances, both non-cytotoxic and cytotoxic DNA-damaging chemicals [213]. It is noted in numerous studies that PCB mixtures produce hepatic lesions [214,215,216,217], which is also documented in other tissues [214,218].

Donato et al. (2021) conducted an analysis that involved patients diagnosed with HCC who had significant serum PCB prior to their diagnosis. The results of the study, however, confirmed a previous hepatic involvement of HBV, HCV and alcohol consumption >60 g/day of 10 years. The data highlighted PCB involvement, which contributed to potential development of HCC in highly industrialized areas [219].

Zani et al. (2013) provided good clinical prognostic variables by testing patients within the industrial area, predisposing PCB’s by investigating serum and fat concentration. The correlation was mitigated not according to demographical, clinical or epidemiological variables and PCB concentration increased linearly with the subject’s age [220]. The article reveals that serum lipid-adjusted PCB concentration is a viable indicator of PCB body storage and that it is substantially linked with PCB concentrations measured in other biological samples.

Notably, the researchers looked into PCB metabolites; PCB29-pQ outlines latent metabolic processes, and particularly focuses its attention on the quinones (recorded as organic pollutants with carcinogenic characteristics and genotoxicity), causing reactive oxygen species (ROS) formation [221,222,223,224,225,226]. Song, Li, et al. (2015) pointed to the level of complexity through biochemical relations, namely the fact that PCB29-pQ promotes S-phase cell proliferation by suppressing cyclins A/D1/E, cyclin-dependent kinases (CDK 2/4/6), and cell division cycle 25A (CDC25A), increasing p21/p27 protein expression. PCB29-pQ also causes apoptosis by upregulating Fas/FasL and activating caspase 8/3 [221].

Norback and Weltman (1985) used a diet-controlled technique to evaluate successive morphologic changes in murine test subjects, and hepatocellular neoplasms were found in 95% of the 47 females and 15% of the 46 males [227]. Another study hypothesized a dose-dependent manner in the formation of HCC, although, interestingly, iron deposition in hepatocytes caused by PCBs is an early occurrence that may be linked to tumor growth. In addition, 52-week exposure to PCB’s was statistically linked with tumor occurrences at termination in both male and female’s specimens across all PCB dose groups [228].

A new study published in the *Journal of Hazardous Material* was concerned with lipidomic analysis, where both single PCB exposure-induced significant lipidomic changes were described [229]. Determining the toxicity of microplastics and the possibility of a “Trojan Horse” impact lipidomic analysis was used on HCC HepG2 cell lines [217,218,219,220,221,222,223,229]. The HepG2 cells analysis revealed that both single PCB exposures generated substantial lipidomic alterations, particularly for glycerophospholipids and glycerolipids, implying a major modification of cell membrane integrity and susceptibility [229]. Menéndez-Pedriza et al. (2022) may have found a potential way to measure an alternative method of analysis for PBC exposure, which must be confirmed by further studies [229].

### 7.4. Pancreatic Involvement

New studies revealed significant involvement of pancreatic cells, as they switch on certain molecular pathways, leading to cytokine signaling paths for inflammation and carcinoma [230]. Lin et al. (2014) studied the effect of Aroclor 1254, which can induce inflammatory stressors and oncogenic changes in Kras and Kras proteins for exocrine pancreatic cancer [230], although it was concluded that it was difficult to identify. What was interesting within this study was the mention of the contributing factors for the activation of phosphorylation in the ERK1/2-P90RSK1-Bad signaling cascade to protect against cell-mediated death.

Nyska et al. (2004) carried out an investigation of chronic exposure to dioxin and dioxin-like compounds in the pancreas in female Harlan Sprague-Dawley rats [231]. Over a 2-year course, they reported induced pancreatic lesions from as low as 100 ng/kg/day of PCB-126, and in a dose-dependent manner, they also reported an increase in the number of lesions, vacuolization of acini, inflammation, and greater risks for adenoma formation. The current study mentioned that serological values were not measured and it would be potentially interesting to investigate the levels of islet-related hormones in the blood and cross examine the results for any significance.

### 7.5. Pancreatic Ductal Adenocarcinoma (PDA)

Porta et al. mentioned potential relationships between known oncogenes such as *Ras* and *KRAS signaling* pathways, although organochlorine compounds (OCs) and coffee consumption may play a role in pancreatic cancer etiology by modulating KRAS activation or maintenance [232,233,234]. This analysis sought to test the hypothesis that in PDA, mechanistic links exist between the concentrations of various OCs and the prevalence of KRAS oncogene mutations at diagnosis, as well as between the latter with coffee consumption. The results were congruent with their hypothesis and its association with PDA and KRAS mutations. KRAS mutations had greater amounts of p,p′-DDT, p,p′ DDE, and PCBs 138, 153, and 180 than instances with wild-type KRAS, although only for p,p′-DDT and PCBs 138 and 153 were the differences statistically significant. It is also worth noting that p,p′-DDT, PCBs, and coffee may all play distinct roles in the etiology of PDA by altering KRAS activation, acquisition, or persistence, perhaps via indirect, non-genotoxic, or epigenetic pathways. Further studies are needed to be conducted and with a larger sample size to enhance the corresponding relationships between OC’s and PDA and other pancreatic neoplasms.

### 7.6. Pancreatic Cancer

A multi-center study conducted an assessment that involved the relationships between occupational history and serum concentrations of OCs in exocrine pancreatic cancer (EPC) [235]. The study presented the framework that could further advance the necessary expertise of the relationships between OCs and serum concentrations. Interestingly, the data extracted from the results demonstrated more PCB interaction with high serum concentrations amongst workers of the metal industry, in contrast to OC serum content, which was significantly lower within agriculture workers. Bosch de Basea et al. (2010) highlighted the need to perform further studies on serum analysis and OCs in identifying key occupational sources of OC contamination in EPC and determining the extent to which OCs may explain the link between specific industries and EPC [235]. The adjusted lipid results obtained from another study that measured OC in serum reported a total of 108 pancreatic cancer cases and 82 control subjects aged 32–85 years. The results highlighted some difficulty, as cachexia belongs to symptoms of pancreatic cancer, and its influence on blood OC levels is difficult to predict. One probable impact of cachexia is OC bio-concentration in the reduced lipid pool, which would result in bias away from the null [236,237].

A small number of studies have mentioned that diet impacts serum OC concentrations and *K-Ras* mutations in EPC; one study pointed to dairy products with the exclusion of butter, showing that the increase in the consumption of milk and other dairy products is definitely linked to higher levels of p,p’-DDT, PCB 138, and PCB 153. The consumption of milk and other dairy products was also shown to be substantially related to blood levels [238]. Amongst other food groups, including meat and fish, an insignificant association was noted as well as an inverse association between consumption of meat and sausages and p,p’-DDT was not significant [238,239].

Correlating K-Ras mutations in cases of EPC possibly provoked a new interest of studies, considering the popularity of daily consumption of dairy products. Morales et al. (2007) explored patients with a K-ras mutations that reported higher intakes of dairy products [238]; however, the presence of OC and pancreatic cancer possibly both play a significant role in the development of neoplastic changes [232,238,239,240,241].

Serum concentrations of p,p’-DDT were considerably greater in pancreatic cancer cases with a K-ras mutation compared to patients without a mutation, according to a comparative study from the PANKRAS II Group [235].

Wider and more extensive research must be performed on whether diet plays a significant role in impacting OC concentration in the development of pancreatic neoplasms across both animal and human models.

### 7.7. Leukemia

Except for solid tumors, micro- and nanoplastics particles might also be a threat regarding the potential onset of liquid malignancies. A recent study by Leslie et al. (2022) proved that plastic particles (primarily polyethylene terephthalate, polyethylene, and polymers styrene, along with poly (methyl methacrylate)) might accumulate in the human bloodstream [242]. However, it is still unknown whether plastic particles can be found in plasma and what specific cell types are responsible for the transporting of plastic particles. Sun et al. (2021) pointed out that polystyrene microplastic induces hematotoxicity and disturbances in metabolic, Jak/Stat, and T cell homeostasis pathways in mice [243]. Furthermore, the researchers indicated that polystyrene microplastic also causes a decrease in the amount of white blood cells in the peripheral blood, as well as the inhibition of the colony-forming ability of the bone marrow cells. The results of these studies suggest that the presence of plastic in the human bloodstream might also induce hematotoxicity; however, this hypothesis must be verified in the future studies (Table 1).

## 8. Conclusions

The actual impact of microplastics and nanoplastics on human health cannot be clearly and completely defined, since it requires extensive, multi-disciplinary long-term research. Undoubtedly, plastic’s carcinogenic/mutagenic impact on cells has already been broadly reported, fueling researchers’ concerns, and thus the willingness to further research this matter. Except being potentially harmful themselves, microplastics might also be contaminated with other substances, including harmful organic chemicals or trace metals, whose exposure to living organisms might be toxic. So far, the knowledge regarding the relationship between the exposure to nano- and microplastics and the onset of carcinogenesis is relatively scarce and has only been investigated with regards to several types of cancer, such as hepatocellular carcinoma or pancreatic cancer, which indicates an alarming need of further, comprehensive research.

## Figures and Tables

**Table 1:** Effect of plastic-related compounds on cancer pathogenesis.

Type of Cancer	Effect on Pathogenesis
Endocrine-related cancers	Endocrine disruptors are chemicals found in many everyday products, including plastic bottles and containers Endocrine disruptors interfere with the synthesis, secretion, transport, binding, action, or elimination of natural hormones in the body Endocrine disruptors may act through estrogen/testosterone coupling receptors
Biliary tract cancer	Endocrine-disrupting agents, with recognized estrogenic activity (alkylphenols, PCB, bisphenol A), increase the risk of extrahepatic biliary tract cancer Cancers were mainly located in the extrahepatic bile duct and ampulla of Vater
Hepatocellular carcinoma	PCB contributes to potential development of HCC Serum lipid-adjusted PCB concentration is a viable indicator of PCB body storage The PCB29-pQ—PCB metabolite causes ROS formation and affects cell cycle by suppressing cyclins A/D1/E and cyclin-dependent kinases, while upregulating Fas/FasL and activating caspase 8/3 PCB exposures generates substantial lipidomic alterations, causing a major modification of cell membrane integrity and susceptibility
Pancreatic cancer	Organochlorine compounds may modulate KRAS activation or maintenance p,p′-DDT and PCBs may all play a role in the etiology of pancreatic ductal adenocarcinoma and exocrine pancreatic cancer by altering KRAS activation
